# Apical periodontitis in osteoporotic postmenopausal women: Systematic review and meta-analysis

**DOI:** 10.4317/medoral.26697

**Published:** 2024-08-01

**Authors:** Victoria Areal-Quecuty, Cristiane Cantiga-Silva, Aurea Simón-Soro, Daniel Cabanillas-Balsera, Jenifer Martín-González, Juan J Saúco-Márquez, Juan J Segura-Egea

**Affiliations:** 1 Department of Stomatology, Section of Endodontics, School of Dentistry, University of Sevilla, Sevilla, Spain; 2 Department of Preventive and Restorative Dentistry, School of Dentistry, São Paulo State University (UNESP), Araçatuba, SP, Brazil

## Abstract

**Background:**

The aim of this systematic review and meta-analysis was to analyze the scientific evidence about the association between osteoporosis in postmenopausal women and the prevalence of apical periodontitis, assessed radiographically.

**Material and Methods:**

PRISMA Guidelines have been followed. The research question was: In adult women, does the presence or absence of osteoporosis affect the prevalence of AP, radiographically diagnosed? A systematic search was performed in PubMed/MEDLINE, Web of Science, Scopus and EMBASE. The meta-analyses were calculated with the Open Meta Analyst software. Risk of bias was assessed using the Newcastle Ottawa Scale. The certainty of evidence was assessed by GRADE.

**Results:**

Four studies were selected. Meta-analysis showed an overall OR = 2.2 (95% CI = 0.94 - 4.97; *p* = 0.07), indicating that osteoporotic women had approximately twice the probability of having periapical lesions, compared to control women, although the difference was only marginally significant. The overall risk of bias of the included studies was moderate, and the certainty of evidence was low.

**Conclusions:**

Apical periodontitis, assessed as periapical lesion, is more frequently diagnosed in osteoporotic women, who are twice as likely to have periapical radiolucent lesions.

** Key words:**Apical periodontitis, endodontic medicine, menopause, meta-analysis, older adults, osteoporosis, postmenopausal women, systematic review.

## Introduction

The progression of the caries lesion, without treatment, causes pulp infection and, finally, pulp necrosis (1). Pulpal infection spreads through the apical foramen causing an inflammatory reaction around the root apex, named apical periodontitis (AP) (2). Chronic apical periodontitis manifested radiographically by the presence of a periapical radiolucent image (3). The prevalence of AP in the general population is very high, with 52% of people having at least one tooth with AP (4). AP is not only a local inflammatory process, but can have systemic implications (5). Moreover, AP has been associated with diabetes (6), cardiovascular diseases (7), metabolic syndrome (8), inherited coagulation disorders (9), inflammatory bowel disease (10,11), chronic kidney disease (12), and chronic liver disease (13).

On the other hand, in postmenopausal women estrogen deficiency provokes osteoporosis, characterized by a decrease in bone mass, with alteration of bone architecture and reduction in bone strength (14). Osteoporosis causes rapid bone loss that mainly affects trabecular bone (15), affecting jaw bones in a similar way to other bones. Osteoporosis has been linked to AP, suggesting that osteoporotic patients could suffer greater periapical bone loss (16), with increasing prevalence of periapical radiolucent lesions.

This systematic review aimed to analyze the scientific literature on the association between AP, diagnosed radiographically, and osteoporosis in postmenopausal women.

## Material and Methods

- Information sources and search strategy

This systematic review is reported in accordance with Preferred Reporting Items for Systematic Reviews and Meta-Analyses (PRISMA) guidelines (17). The study protocol was registered at PROSPERO: CRD42023494749.

A literature examination was carried out to identify the articles related to AP, diagnosed radiographically, comparing osteoporotic women with healthy controls. The research was conducted in PubMed, SCOPUS and EMBASE, using combining Medical Subject Heading (MeSH) terms and text word. A complementary screening on the references of the selected studies was performed. The search strategy included the following key words: (apical periodontitis OR endodontics OR periapical disease OR periapical granuloma OR periapical periodontitis) AND osteoporosis AND (prevalence OR frequency).

- Eligibility criteria

A specific research question was structured according to PICO method: In adult women (Population), does the presence of osteoporosis (Intervention / Condition), compared to its absence (Comparison), affect the prevalence of AP, diagnosed radiographically (Outcome)?

The inclusion criteria established were articles published between January 1980 to December 2023, reporting a) clinical epidemiological studies, b) studies comparing women with osteoporosis and normal premenopausal women, and c) studies providing data on the prevalence of apical periodontitis, assessed radiographically, both in patients with osteoporosis and normal control women. Exclusion criteria were defined as a) studies carried out in animals or in cell culture, b) studies reporting data only from osteoporotic women, c) studies that did not report information about the prevalence of AP. Articles that did not meet the inclusion criteria were excluded. In vitro studies, animal studies, case series, studies reporting data only from postmenopausal women, and studies reporting only clinical data, without radiographic diagnosis of AP, were excluded.

- Study selection and data collection

The bibliographic search and the screening of articles was performed by two independent reviewers (JJS-E and VA-Q). The titles and abstracts of the retrieved references were screened for relevance and after this, the full texts of all articles potentially eligible were analyzed against the inclusion/exclusion criteria. Any disagreement was resolved between them or by third reviewer (AS-S) to minimize risk of bias.

The methodology of selected studies was examined, and main features were extracted and compiled including, authors, date of publication, study design, subjects and sample size, main quantitative results and odds ratio values, and type of radiograph for the diagnosis of periapical lesions. The main outcome was the prevalence of AP assessed radiographically.

Data extraction was performed by three investigators JJS-E, VA-Q and AS-S. Disagreements were resolved by discussing between the three and reaching an agreement by majority. When necessary to clarify the data, the authors of the included studies were consulted.

- Risk of bias assessment

The risk of bias of the included studies was assessed using the Newcastle-Ottawa Scale (18), adapted for cross-sectional studies (19). This scale was adapted to the outcome of interest, classifying the items into two domains: sample selection and outcome. They were given points (*) depending on the aspect required were present or missing. Three authors (VA-Q, DC-B, and JJS-E) assessed the risk of bias of each of the included studies independently. In case of disagreement, the authors discussed until they reached an agreement. Two domains, sample selection and outcome, were considered. The evaluation of each item was made according to the following criteria.

A) Domain “Sample selection” (maximum: six points):

1) Representativeness of the sample: Truly representative of the average in the target population (random sampling): three points; Somewhat representative of the average in the target population (non-random sampling): two points; Selected group of users: one point; No description of the sampling strategy: no points.

2) Sample size: Justified, the study provided sample size calculation, or the entire population was recruited (and the loss rate was ≤20%): one point; Not justified size: no points.

3) Osteoporotic condition: The osteoporotic condition was verified by means of bone mineral density: two points; osteoporotic condition was established only by post-menopausal status: one point; The condition of osteoporosis was established only by age or no criteria description: no points.

B) Domain “Outcome” (maximum: six points):

1) Assessment of the outcome: Training and calibration for the methodology of assessing radiographically periapical lesions with inter- and intra-agreement values provided: two points; Training and calibration for the methodology of assessing radiographically periapical lesions, with inter- or intra-agreement values not provided: one point; Training and calibration not mentioned: no points.

2) Type of radiographs used: Periapical radiographs: two points; panoramic radiographs: one point; the type of radiography used is not specified: no points.

3) Inclusion of third molar in the total sample of teeth: Third molar included: one point. If the study did not mention that third molar was excluded, it got one point in this domain; third molar not included: no points.

4) Number of observers: Radiographs were studied by two or more examiners: one point; only one examiner studied the radiographs: no points.

The maximum possible score was 12 points. High risk of bias was defined as 0 to 4 points, moderate risk of bias was considered for the studies scoring 5 to 8 points, and finally low risk of bias was assigned to studies scoring between 9 and 12 points.

- Statistical analysis

To determine the pooled OR and its 95% CI, the random-effect model meta-analysis was performed using the OpenMeta Analyst (20), version 10.10 software. The primary outcome was the prevalence of AP, so the variables analyzed were the prevalence of AP in osteoporotic women and normal premenopausal women. Forest plots were made to graphically represent the odds ratio of AP in both menopausal and premenopausal women. The level of significance was applied to *p* = 0.05.

To estimate the variance and heterogeneity amongst trials, the Higgins I2 test were employed, considering a slight heterogeneity if it is between 25 and 50%, moderate between 50 and 75%, and high if >75% (21).

- Grading of recommendations assessment, development and evaluation

The Grading of Recommendations Assessment, Development, and Evaluation (GRADE) tool was used to assess overall certainty of evidence (22,23). Three investigators (VA-Q, JJS-E, and DC-B) independently carried out the assessment.

## Results

- Bibliographic search

**Figure 1:**
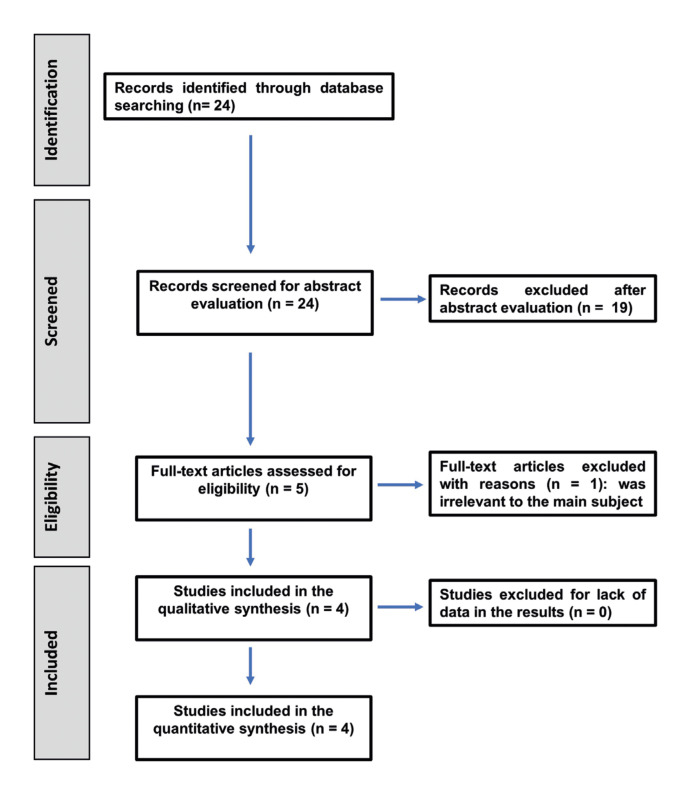
Flowchart on the search strategy carried out according to PRISMA 2020 indications.

The flow diagram of literature search strategy and selected studies for this review is shown in Fig. 1, according to PRISMA 2020 instructions (17). Initial search of different databases resulted in twenty-four published studies, with no duplicates.

Then, of the twenty-four eligible papers, after analysing the titles and abstracts, nineteen that did not fulfil the inclusion criteria were excluded, selecting five for reading the full text. After comprehensive reading, one study was excluded because it was irrelevant to the main subject (24).

- Characteristics of the included studies

Finally, four studies were selected for the systematic review and meta-analysis (16,25-27). The four studies were cross-sectional studies comparing the prevalence of AP in postmenopausal women with that of healthy premenopausal control women. The main characteristics of the included studies, i.e. study design, study sample, method for diagnosis of AP, method for diagnosis of osteoporotic state, and main results, are summarized in Table 1.

- Meta-Analysis

Data from selected articles were analyzed and summarized in an evidence Table containing the data, descriptive statistics, and ORs calculated (Table 2). The four studies added a total of 932,949 people, of which 5,360 (0.6%) have periapical lesions. The estimated variance between studies was examined using the Tau2 test and was found to be significant (Tau2 = 0.53; Q = 17.461 df = 3; *p* < 0.01).

The evidence for heterogeneity (I² = 83%; *p* < 0.01) was very high. Therefore, the weights of each study were calculated using the random effects model, considering that there was variation between the included studies and allowing the study results to vary in a normal distribution.

**Figure 2:**
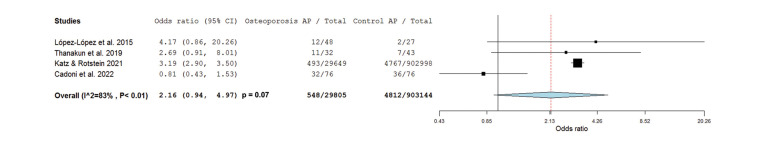
Forest plot of the included studies in the meta-analysis for the prevalence of apical periodontitis.

Fig. 2 shows the forest plot of the meta-analysis, the ORs of each study and the pooled OR, calculated using the DerSimonian-Laird method with random effects.

Among osteoporotic women, 548 (1.8%) had AP, while in healthy controls 4,812 showed periapical lesions (0.5%). The overall OR was resulting in an OR = 2.2 (95% CI = 0.94 - 4.97; *p* = 0.07), indicating that osteoporotic patients were more than twice the probability of having periapical lesions, compared to control women, but this difference was only marginally significant.

- Publication bias

Publication bias could not be assessed quantitatively as there were fewer than the required minimum of 10 studies (21).

- Risk of bias assessment

According to the Newcastle-Ottawa Scale (18), risk of bias was evaluated for each study (Table 3). One study was classified as low risk of bias (16), another study was considered as moderate risk of bias (27), and two other studies were classified as high risk of bias (25,26). The overall sum of the scores of the 4 studies was 25, indicating a moderate overall risk of bias.

- GRADE evaluation: level of certainty

The certainty of evidence was rated using the GRADE tool (Table 4). The four studies were observational cross-sectional studies. The domain risk of bias, according to its overall result, was classified as “not serious”. The domain inconsistency received the ‘serious’ classification as the heterogeneity was high (I2 = 83%). Indirectness domain was classified as ‘not serious’ as the studies did not perform indirect comparisons or present indirect results. The included populations were representative of the osteoporotic women and AP was reliably evaluated. However, the domain of imprecision was rated ‘serious’ since the 95% CI of the estimated effect (OR) was out of 0.75-1.25, and the magnitude of the number of studies included was moderate (< 5 studies). While there were not enough studies to perform a quantitative assessment of publication bias, it was not considered significant enough to downgrade the quality of evidence, as studies from various journals with varying sample sizes were included and none were funded by the private sector. For all the above, the certainty of the evidence was classified as low, indicating that the true effect might be markedly different from the estimated effect.

## Discussion

This systematic review aimed to analyse the available evidence about the association between osteoporosis and the prevalence of AP radiographically diagnosed. The results showed that postmenopausal women with osteoporosis were twice as likely (OR = 2.2) to show periapical radiolucent lesions, compared to control healthy women. This OR value implied a high strength of association between both variables, although the *p value* was marginally significant (*p* = 0.07).

In recent decades, the search for possible interrelationships between systemic health and endodontic variables (5,28), including apical periodontitis and root canal treatment (29), has been carried out through epidemiological studies, mostly cross-sectional studies. This is the case for osteoporosis. The hypothesis of the existence of an association between systemic health and apical periodontitis requires the demonstration of biological mechanisms that connect them. For osteoporosis, some biological mechanisms can be suggested by which the periapical status could be affected. The alteration of the bone remodeling process of maxillary bones secondary to estrogen deficiency, could exacerbate a pre-existing inflammatory condition, such as chronic apical periodontitis, causing aggravated resorption (30).

Experimentally, ovariectomy enhanced alveolar bone loss in rats with periodontitis, indicating that postmenopausal osteoporosis may influence the progression of periodontitis (31), and osteoporosis has been also associated with the onset and progression of periodontal disease in humans (32,33). Moreover, low bone mineral density in postmenopausal women has been showed to be associated with a higher frequency of radiolucent periapical lesions (16). Estrogen deficiency in postmenopausal women would increase inflammatory bone resorption in the periapical lesions, resulting in a higher net bone loss, with the consequent intensification of periapical radiolucency, which would facilitate its radiological detection (26). Researchers have demonstrated that postmenopausal women experience a more rapid decrease in alveolar bone density, indicating that estrogen deficiency affects bone tissue in the jaws (34). It has been reported a decrease in bucco-lingual alveolar thickness, possibly due to periosteal resorption, in post-menopausal women with low bone mineral density (35). Furthermore, mandibular alveolar bone mass and the degree of trabeculation correlate with skeletal bone mineral density (36).

Taken together all the results mentioned above suggest that the changes produced by osteoporosis in alveolar bone in postmenopausal women would increase the probability of radiographycally detect the radiolucent lesion of AP. This would be one of the explanations for the higher prevalence of AP in postmenopausal women. However, it cannot be ruled out that the greater resorption of bone trabeculae in the jaws of postmenopausal women facilitates the production of false positives when the periapical region is evaluated radiologically (16).

The result of this systematic review should be evaluated with caution, taking into account the limitations of the study. The strength of the association between osteoporosis and the prevalence of AP (OR = 2.2) could, at first glance, be considered a strength of the study, but the *p value* (*p* = 0.07) and the wide 95% confidence interval (out of 0.75-1.25), prevent doing so.

Publication bias could not be quantitatively assessed since the number of studies included in the meta-analysis did not reach the required minimum of 10 (21). However, it should be noted that none of the studies included in the meta-analysis were funded by the private sector. None of the authors reported any type of conflict of interest.

Indirectness was considered not serious, since all four studies used appropriate diagnostic methods for both apical periodontitis and osteoporosis. However, the fact that panoramic radiographs were used in several of the studies (16,25) to evaluate the periapical status can also be considered a limitation of the present systematic review. It has been reported that an underestimation of lesions occurred when panoramic radiography was used (37), but the difference with periapical radiography was not statistically significant (38). Nevertheless, among the criteria to assess the risk of bias, the type of radiography used to diagnose AP has been considered. On the other hand, none of the included studies controlled the possible bias that hormonal replacement therapy could produce, since some of the women included in the osteoporotic group would surely be taking it.

Regarding the overall risk of bias assessment, it was carried out according to the Newcastle-Ottawa Scale (18), indicating a moderate risk of bias, so it was considered not serious. However, the assessment of the quality of the overall evidence using GRADE showed that the level of certainty is low. Therefore, it is likely that the real effect is substantially different from the estimated effect, i.e. OR = 2.2 (*p* = 0.07). The contribution of new studies will help to clarify and quantify more precisely the possible association between osteoporosis and the prevalence of AP.

In the GRADE evaluation, the high heterogeneity (I2 = 83%) has also been considered, so the inconsistency was assessed as serious. For this reason, the random effects model has been used to carry out the meta-analysis.

Finally, it should be noted that the result of this study cannot be interpreted in terms of causality (6). The four studies included are cross-sectional, so we can only speculate on the possible association between osteoporosis and the prevalence of AP. Although possible biological mechanisms of interrelation between both conditions have been previously suggested, it has been done as a matter of speculation, never as causal mechanisms.

The results of the present study should be transferred to the daily practice of dentistry. Considering the large number of postmenopausal women seen in the dental clinic, the dentist should know that the probability of them suffering from periapical disease is greater, so they should pay more attention to the diagnosis of periapical lesions in these patients. Definitively, dentists should collaborate with physicians treating postmenopausal women, and the evaluation of the periapical status should be part of the routine clinical control of these patients, especially those with low bone densitometry values.

## Conclusions

Periapical lesions are more frequently diagnosed in osteoporotic women, who are twice as likely to have AP. The dentist's evaluation of the periapical status should be part of the routine clinical control of postmenopausal women, especially those with low bone densitometry values. Prospective studies with large samples are necessary to determine more precisely the extent of the association between periapical disease and osteoporosis.

## Figures and Tables

**Table 1:** Summary of descriptive characteristics of the included studies.

Authors	Study design	Study sample	Diagnosis of apical periodontitis	Diagnosis of osteoporosis	Association osteoporosis / apical periodontitis
López-López *et al.* 2015	Cross-sectional	Controls: 27 Osteoporotic: 48	Panoramic radiographs	Bone densitometry	No; p > 0.05
Thanakun *et al.* 2019	Cross-sectional	Controls: 43 Post-menopausal: 32	Panoramic radiographs	Menopause	No; p > 0.05
Katz and Rotstein 2021	Cross-sectional	Controls: 902,998 Osteoporotic: 26,649	Diagnostic code ICD 9-522.5 or ICD 10-K04.7	Diagnostic code ICD 9-733 or ICD10-M81	Yes; p < 0.05
Cadoni *et al.* 2022	Cross-sectional	Controls: 76 Post-menopausal: 76	Periapical radiographs	Bone densitometry	No; p > 0.05

**Table 2:** Extracted data and ORs calculated for each one of the included studies.

Authors and year	Pre-menopausal control women		Post-menopausal osteoporotic women		OR (95% C.I.)	p
	AP / Total	AP (%)	AP / Total	AP (%)		
López-López *et al.* 2015	2/27	7.4	12/48	25.0	4.2 (0.9 - 20.3)	0.06
Thanakun *et al.* 2019	7/43	16.3	11/32	34.4	2.7 (0.9 - 8.0)	0.07
Katz and Rotstein 2021	4,767/902,998	0.5	493/29,649	1.7	3.2 (2.9 - 3.5)	0.00
Cadoni *et al.* 2022	36/76	47.4	32/76	42.2	0.8 (0.4-1.5)	0.51
TOTAL	4,812 / 903,144	0.5	548 / 29,805	1.8	2.2 (0.9-5.0)	0.07

AP: apical periodontitis. OR: odds ratio.

**Table 3:** Risk of bias of individual studies assessed using the Newcastle-Ottawa Scale for assessing risk of bias. The maximum possible score was 12 points (48 points for the four studies). High risk of bias was defined as 0 to 4 points, moderate risk of bias was considered for the studies scoring 5 to 8 points, and finally low risk of bias was assigned to studies scoring between 9 and 12 points.

Studies	Sample selection			Outcome				Risk of bias
	Representativeness of the sample (max: 3)	Sample size calculation (max: 1)	Osteoporotic condition (max: 2)	Assessment of the outcome (max: 2)	Type of radiograph (max: 2)	Inclusion of third molar (max:1)	No. Of observers (max:1)	
López-López et al 2015	**	*	**	**	*	-	*	9 (Low)
Thanakun *et al.* 2019	**	-	*	-	*	-	-	4 (High)
Katz and Rotstein 2021	**	-	**	-	-	-	-	4 (High)
Cadoni *et al.* 2022	**	-	**	**	*	-	*	8 (Moderate)
OVERALL	8	1	7	4	3	0	2	25 (Moderate)

**Table 4:** Grade assessment of certainty level.

Certainty assessment							Certainty	Importance
No. of studies	Study design	Risk of bias	Inconsistency	Indirectness	Imprecision	Other considerations		
Osteoporosis - apical periodontitis								
4	observational studies	not serious^a^	serious^b^	not serious	serious^c^	OR: 2.16 (0.94-4.97) p = 0.07	⊕⊕⊙⊙ LOW	IMPORTANT

Gade Working Group grades of evidence: Explanations: a. Detailed in table 3: Risk of bias summary (moderate) b. I^2^ = 83% (p < 0.01) c. 95% CI out of 0.75-1.25 High certainty: The authors have a lot of confidence that the true effect is similar to the estimated effect Moderate certainty: The authors believe that the true effect is probably close to the estimated effect Low certainty: The true effect might be markedly different from the estimated effect Very low certainty: The true effect is probably markedly different from the estimated effect.
